# CNTF Mediates Neurotrophic Factor Secretion and Fluid Absorption in Human Retinal Pigment Epithelium

**DOI:** 10.1371/journal.pone.0023148

**Published:** 2011-09-02

**Authors:** Rong Li, Rong Wen, Tina Banzon, Arvydas Maminishkis, Sheldon S. Miller

**Affiliations:** 1 National Eye Institute, National Institutes of Health, Bethesda, Maryland, United States of America; 2 Bascom Palmer Eye Institute, University of Miami, Miller School of Medicine, Miami, Florida, United States of America; Johns Hopkins University, United States of America

## Abstract

Ciliary neurotrophic factor (CNTF) protects photoreceptors and regulates their phototransduction machinery, but little is known about CNTF's effects on retinal pigment epithelial (RPE) physiology. Therefore, we determined the expression and localization of CNTF receptors and the physiological consequence of their activation in primary cultures of human fetal RPE (hfRPE). Cultured hfRPE express CNTF, CT1, and OsM and their receptors, including CNTFRα, LIFRβ, gp130, and OsMRβ, all localized mainly at the apical membrane. Exogenous CNTF, CT1, or OsM induces STAT3 phosphorylation, and OsM also induces the phosphorylation of ERK1/2 (p44/42 MAP kinase). CNTF increases RPE survivability, but not rates of phagocytosis. CNTF increases secretion of NT3 to the apical bath and decreases that of VEGF, IL8, and TGFβ2. It also significantly increases fluid absorption (*J*_V_) across intact monolayers of hfRPE by activating CFTR chloride channels at the basolateral membrane. CNTF induces profound changes in RPE cell biology, biochemistry, and physiology, including the increase in cell survival, polarized secretion of cytokines/neurotrophic factors, and the increase in steady-state fluid absorption mediated by JAK/STAT3 signaling. *In vivo*, these changes, taken together, could serve to regulate the microenvironment around the distal retinal/RPE/Bruch's membrane complex and provide protection against neurodegenerative disease.

## Introduction

Ciliary neurotrophic factor (CNTF) was discovered in chicken embryo extract where one third of its neurotrophic activity originated in the eye [1]. It has demonstrated a remarkable capacity for neuroprotection of rod photoreceptors in numerous retinal degeneration models across several species [2], [3], [4], [5], [6], [7]. Recently, CNTF was found to promote cone outer segment regeneration and protect cone photoreceptors [8]. In addition, retinal injuries induce upregulation of CNTF, which is thought to protect photoreceptors [9], [10], [11]. These studies led to several CNTF clinical trials for retinal degenerative disorders. In a phase I clinical trial, CNTF-secreting implants (ECT) resulted in improved visual acuity in several patients with late stages of retinitis pigmentosa (RP) [12]. A recent clinical trial found that CNTF implant stabilizes vision loss in patients with geographic atrophy [13]. CNTF implants also prevent secondary cone degeneration in patients with RP [14].

**Figure 1:**
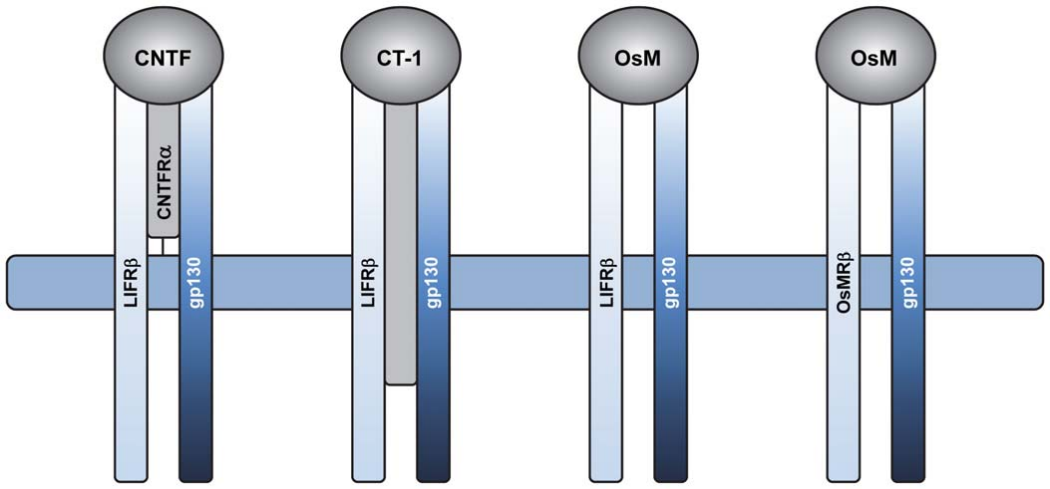
Schematic diagram of CNTF, CT1 and OsM cytokine receptor complexes. The cytokines are depicted schematically as grey circles. Signal transducing-receptor subunits are light blue (LIFRβ and OsMRβ) or dark blue (gp130). α-receptor subunits are shown in light grey.

CNTF is a member of the interleukin-6 (IL6) family of neuropoietic cytokines that includes IL11, leukemia inhibitory factor (LIF), oncostatin M (OsM), and cardiotrophin 1 (CT1) [15]. It binds to a receptor complex of three components: CNTF receptor α (CNTFRα) and two signal-transducing transmembrane subunits, LIF receptor β (LIFRβ) and gp130. The schematic diagram in Figure 1 shows that LIFRβ/gp130 heterodimers are shared by CNTF, CT1, and OsM, which can also signal through gp130/OsMRβ receptor complex [16]. Previous studies suggested the absence of LIFRβ in RPE resulting in a lack of responsiveness to CNTF (Song Y, et al. *IOVS* 2003;44:ARVO E-Abstract 390). However, the development of a human culture model of RPE that closely mimics native human adult RPE and the tight anatomical relationship between RPE and photoreceptors prompted us to reexamine the role of CNTF in RPE physiology. The RPE is a monolayer of polarized epithelial cells strategically located between the photoreceptors and the choroidal blood supply in the posterior part of the eye. It selectively transports ions, fluid, nutrients, and metabolic waste products between the neuronal retina and the choriocapillaris [17] and helps maintain the health and integrity of the distal retina by regulating the chemical composition and volume of the extracellular spaces that face the RPE apical and basolateral membranes [18], [19], [20]. Equally important are its roles in the visual cycle [21] and in photoreceptor outer segment renewal [22].

In the present study, we used primary cultures of hfRPE to understand how CNTF can regulate RPE function. These cultures molecularly and physiologically mimic native human tissue [17], [18], [19], [23], [24], [25]. We report here that the apical membrane of hfRPE expresses all of the CNTF receptor subunits mediating binding and signal transduction. Activation of these receptors increases RPE survival. In addition, CNTF increases active, ion-linked fluid absorption across the RPE while modulating the polarized secretion of neurotrophic factors and cytokines to the apical bath. All of these responses, taken together, likely contribute to the protective effect of CNTF on photoreceptors [14], [26], [27].

## Materials and Methods

### Ethics Statement

The research adhered to the tenets of the Declaration of Helsinki and the NIH Institutional Review Board. This research was reviewed and approved as an exemption from human subjects (approval # 4426) by the National Institutes of Health Institutional Review Board. Tissues were obtained with the written informed consent of anonymised donors.

### Cell cultures

Human fetal retinal pigment epithelial (hfRPE) cells were isolated from eyes obtained from Advanced Bioscience Resources (Alameda, CA) and cultured in Primaria® tissue culture flasks (BD Biosciences, Franklin Lakes, NJ) in culture medium based on MEM-α, as described previously [23]. Culture medium was changed every 3 days and cells were subcultured by trypsin-EDTA treatment. Cells of passages 1 and 2 were used for all studies. ARPE-19 (transformed cell lines from adult human RPE) was purchased from ATCC Bioresource Center (Manassas, VA) and grown in DMEM/F12 medium (Invitrogen, Carlsbad, CA) containing 10% fetal bovine serum (Atlanta Biologicals, Lawrenceville, GA).

Recombinant human CNTF, CT1, and OsM, with a 6×His tag fused to the N terminus, were expressed in E.coli (XL-blue, Stratagene, La Jolla, CA) and purified under native conditions by immobilized-metal affinity chromatography on Ni-NTA Agarose columns (Qiagen), as described previously [11], [28], [29]. Eluted protein was buffer-exchanged to phosphate buffered saline and stored at −80°C.

### Real-time quantitative PCR

Total mRNA was extracted from confluent monolayers of hfRPE cultured on transwells using the RNeasy Kit (Qiagen, Valencia, CA), according to manufacturer's instructions. Total RNA (1 µg) was reverse transcribed to cDNA with SuperScript III Fist-Strand Synthesis System for RT-PCR (Invitrogen). Quantitative real-time PCR was performed using Taq-Man assays (Applied Biosystems, Forster City, CA) for CNTF, CT1, OsM, CNTFRα, LIFRβ, gp130, OsMRβ, and GAPDH (glyceraldehyde 3-phosphate dehydrogenase, whose amount was used as an internal control) (ABI Sequence Detection System 7900; Applied Biosystems). Levels of mRNA for each gene were normalized to that of GAPDH. Data were obtained using tissues from four different donors (three technical replicates for each gene).

### Immunoblotting analyses

Confluent monolayers of primary hfRPE cells grown for 6–8 weeks in flasks were homogenized in buffer containing 250 mM sucrose, 10 mM Tris-HCl, 10 mM MgCl_2_, and 1 mM CaCl_2_, (pH 7.4) supplemented with proteinase inhibitor cocktail (Roche, Indianapolis, IN). Cell lysates were centrifuged at 900 g for 10 minutes, 10,000 g for 5 minutes and 18,000 g for 20 minutes at 4°C. Collected supernatant was centrifuged at 100,000 g for 1 hour at 4°C and plasma membrane fraction pellet was collected. Protein was quantified by BCA protein assay (Pierce Biotechnology, Rockford, IL).

Enriched membrane proteins (15 µg) were electrophoresed on a NuPAGE® 3–8% Tris-Acetate Gel (Invitrogen) and electroblotted onto nitrocellulose membranes using XCell II™ Blot Module (Invitrogen). Membranes were incubated in StartingBlock™ T20 (TBS) (Pierce Biotechnology) and probed with antibodies against CNTFRα (ab58560, Abcam, Cambridge, MA), LIFRβ (AF-249-NA, R & D Systems, Minneapolis, MN), gp130 (3732, Cell Signaling Technology, Danvers, MA), or OsMRβ (MAB4389, R & D Systems). Human brain membrane lysate (adult, normal tissue) was used as the positive control for CNTFRα, and whole cell lysates of Hela and NIH3T3 cells were used as the positive control for gp130. Membranes were then incubated with horseradish peroxidase (HRP) conjugated secondary antibodies (Pierce Biotechnology) and target proteins were visualized by chemiluminescent substrate (Supersignal West Dura, Pierce Biotechnology). Blot membranes were imaged and quantified by measuring relative intensities using the Autochemie™ system (UVP, Upland, CA) using LabWorks™ Image Acquisition and Analysis software.

For detection of STAT3 and ERK1/2 (p44/42 MAP kinase) phosphorylation, confluent monolayers of hfRPE or ARPE-19 cells were serum starved for 24 hour in serum free medium (SFM). CNTF, CT1, or OsM (10–50 ng/ml) was added to the culture medium and cells were harvested at various time points after treatment. For blocking experiments, cells were pretreated with JAK inhibitor I (5 µM; EMD Biosciences, Gibbstown, NJ) for 1 hour before addition of neurotrophic factors. Cells were lysed with RIPA lysis buffer (Sigma-Aldrich, St. Louis, MO) containing protease inhibitor cocktail and Halt™ phosphatase inhibitor cocktail (Pierce Biotechnology), centrifuged at 14,000 g, and supernatant was collected. Concentration of total protein was determined by BCA protein assay (Pierce Biotechnology). Total protein (40 µg) from each sample was electrophoresed on a NuPAGE® 4–12% Bis-Tris Gel (Invitrogen) and electroblotted onto nitrocellulose membranes. Membranes were blocked and probed with antibodies against STAT3, phospho-STAT3 (Tyr705), ERK1/2, phospho-ERK1/2 (Thr202/Tyr204) MAP kinase (Cell signaling Technology), and GAPDH (for loading control, Abcam).

### Immunofluorescence localization

Confluent human fetal RPE cells were maintained for 6–8 weeks in transwells (polyester membrane, 0.4 µm pore size; Corning Costar, Corning, NY) before experiments. Cells were fixed with 4% formaldehyde, permeabilized with 0.2% Triton X-100 (Sigma-Aldrich), and blocked with a signal enhancer (Image-iT FX, Invitrogen). RPE monolayers were incubated with antibodies against CNTFRα, LIFRβ, gp130, OsMRβ, and ZO-1, labeled with different fluorophores using the Zenon Labeling Technology (Invitrogen) following manufacturer's instructions [30], [31]. Normal mouse, rabbit, or goat serum pre-labeled with fluorophore was used as the negative control. Samples were mounted on glass slide with antifade reagent containing DAPI (Prolong Gold; Invitrogen) and imaged with a Zeiss Axioplan 2 microscope with apotome and Axiovision 3.4 software (Carl Zeiss AG, Germany).

### Cell proliferation assay

Cell proliferation was measured by the BrdU (bromodeoxyuridin) incorporation assay [30]. Human fetal RPE cells were seeded in 96-well Primaria® culture plates at a density of 2.5×10^3^ cells/well and allowed to attach for 24 hours, serum starved in SFM for another 24 hours, and then replenished with SFM or SFM containing CNTF, CT1, or OsM at various concentrations (0.6–80 ng/ml) or medium containing 5% FBS, and incubated for 48 hours. BrdU labeling solution was then added and the cells were incubated for another 24 hours. Cell proliferation rates were assessed using the Cell Proliferation ELISA BrdU Kit (Roche), according to manufacturer's instructions. Six replicates were used for each treatment and the experiments were repeated using cell cultures from two donors.

### Cell survivability assay

For survival assays, hfRPE cells were seeded in 24-well Primaria® tissue culture plate at a cell density of 2.5×10^4^ cells/well and allowed to grow for 3 days. Cells were then washed and cultured in SFM or SFM containing series concentrations of CNTF, CT1 or OsM (0.1–100 ng/ml) for 8 days or 10 days. Quadruplicates were used for each condition. Cell survivability was determined by 3-(4,5-dimethylthiazol-2-yl)-2,5-diphenyltetrazolium bromide (MTT) colorimetric assays, which measures the reduction of yellow MTT by mitochondrial succinate dehydrogenase. Since reduction of MTT can only occur in metabolically active cells, the level of activity is a measure of cell viability. Briefly, MTT labeling solution (Roche) was added to the culture medium for 4 hours, then the medium was aspirated, and solubilization solution was added to each well. Plates were allowed to stand overnight and each sample was measured at 580 nm (reference wavelength: 700 nm) using a spectrophotometric microplate reader (Safire 3; Tecan Trading AG, Mannendorf, Switzerland).

### Photoreceptor outer segment (POS) preparation and phagocytosis assay

Bovine POS were isolated under dim red light from fresh bovine eyes obtained from a local slaughterhouse (J W Treuth & Sons Inc., Baltimore, MD) according to the methods of Molday et al. [32], [33] and labeled with fluorescein isothiocyanate (FITC; invitrogen). Briefly, POS were resuspended in 1 mg/ml FITC dye in 0.1 M Na-bicarbonate (pH 9.0) for 1 hour in the dark at room temperature. Labeled POS were washed twice in 10% sucrose, 20 mM Na-phosphate, pH 7.2, and twice in 2.5% sucrose in MEM and then resuspended in 2.5% sucrose in MEM at 5×10^7^ POS/ml.

The phagocytosis assay was performed according to the methods of Finnemann et al. [34], [35]. Confluent monolayers of hfRPE cultured on transwell filters were fed with FITC-conjugated POS at a ratio of 5 OS/cell for 4 to 6 hours and rinsed four times with PBS containing 1 mM MgCl_2_ and 0.2 mM CaCl_2_ (PBS-CM). To quench FITC fluorescence of surface-bound but not engulfed POS, cells were treated with 0.2% trypan blue for 10 minutes prior to fixation. Cells were fixed by incubation in ice cold methanol for 5 minutes followed by incubation in 4% paraformaldehyde in PBS-CM for 10 minutes at room temperature and stained with Hoechst 33342. Samples with or without trypan blue treatment were scanned using a plate reader (FITC: 495 nM/517 nM; Hoechst 33342: 345 nm/455 nm, respectively) with a Safire 3 spectrophotometric microplate reader; Relative fluorescence values were used to represent total (bound plus internal) and internal POS, respectively. Data are presented as fluorescence intensity ratios which were calculated by dividing FITC dye fluorescence counts by Hoechst 33342 counts, thereby normalizing for the number of hfRPE cells.

### Cytokine secretion assays

Secretion of a variety of growth factors, including β-nerve growth factor (β-NGF), brain-derived neurotrophic factor (BDNF), CNTF, glial cell derived neurotrophic factor (GDNF), neurotrophin-3 (NT3), LIF, IL6, basic fibroblast growth factor (bFGF), vascular endothelial growth factor (VEGF), pigment epithelium-derived factor (PEDF), IL8, monocyte chemotactic protein-1 (MCP1), insulin-like growth factor-binding protein-1 (IGFBP1) and transforming growth factor-β2 (TGFβ2) were measured in confluent monolayers of hfRPE cultured on transwells. Cells were washed with SFM and cultured in SFM containing 10 or 50 ng/ml CNTF for 48 hours. Media from both the apical and basal compartments were collected and stored at −80°C. Levels of the factors were measured by multicomplex profiling ELISA (SearchLight, Pierce Biotechnology) as described previously [18].

### Fluid transport measurements

**Figure 2:**
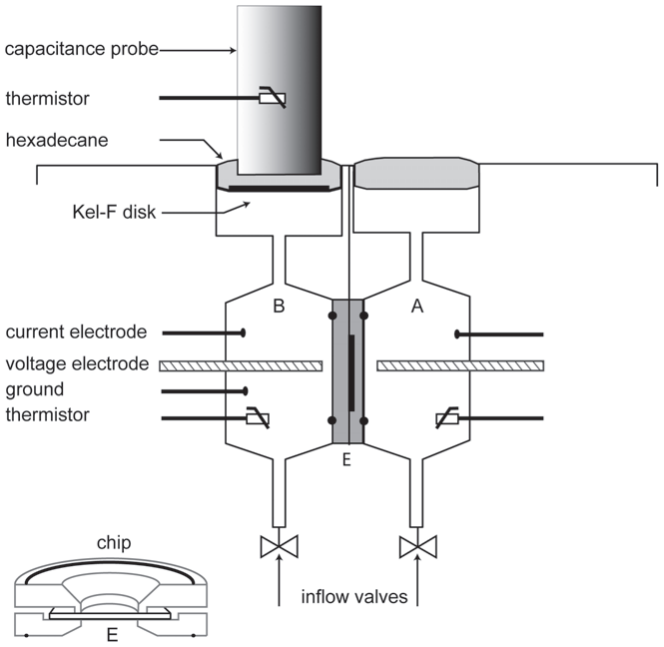
Schematic diagram of fluid transport chamber. Top part of figure shows internal chamber design consisting of two identical hemichambers. Each hemichamber has a voltage-sensing electrode, three current-passing electrodes, and a thermistor. Hemichamber A contains media bathing the apical or retinal surface; hemichamber B contains solution bathing the basal or choroidal surface. Surface of each bathing solution is covered with hexadecane to eliminate evaporation. A capacitance probe is immersed in the hexadecane in one hemichamber. Volume change is detected by changes of distance between probe and bathing media surface. Solutions are exchanged by gravity flow from reservoirs through inflow valves at the bottom and suction from the surface on top. A thin Kel-F disc (dark horizontal line) is used to isolate probe tip from media and avoid short circuit. RPE monolayer (E) is mounted between two circular Kel-F discs with O-rings (shown in lower left corner of the figure). This chip is mounted between two water-jacketed Kel-F blocks in main chamber located in a modified CO_2_ incubator (5% CO_2_, 36.5°C) for fluid transport (*J*v) measurements.

Confluent monolayers of hfRPE cultured on transwells were mounted in a modified Ussing chamber and transepithelial water flow (*J*_V_) measurements were made using a capacitance probe technique, as described previously [18], [19], [36], [37], [38]. As illustrated in the schematic shown in Figure 2, the hfRPE apical and basolateral membranes are physically isolated and the chemical composition of the solutions bathing each membrane can be independently controlled [39], [40]. In addition, tissue integrity and transport capacity were monitored by concomitant recording of transepithelial potential (TEP) and total tissue resistance (R_T_). Steady-state *J*_V_, TEP, and R_T_ were recorded for 20–30 minutes after addition of CNTF to the apical or basal baths. A cAMP-activated Cl channel inhibitor (CFTRinh-172) or a JAK inhibitor I (EMD Biosciences) was used to block the changes of *J*_V_ induced by CNTF.

### Statistical Analysis

Values are means ± SE. Data were analyzed using the Student's *t*-test and are considered statistically significant if *P*<0.05.

## Results

### Expression of CNTF, CT1, OsM, LIF, and localization of their receptors in hfRPE

**Figure 3:**
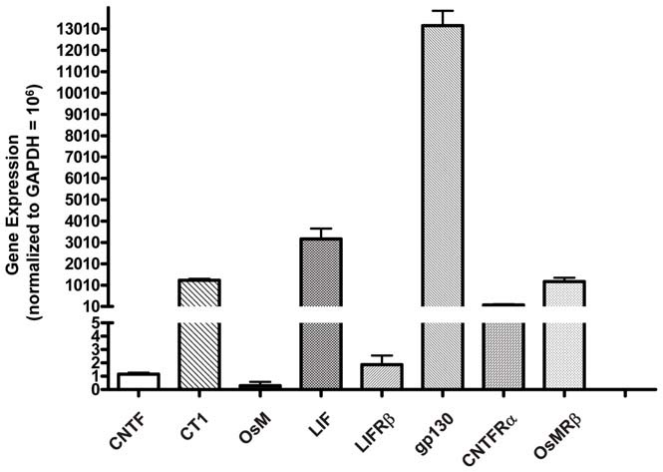
Gene expression of CNTF, CT1, OsM, LIF and their receptors in hfRPE. Relative amounts of messenger RNA were quantified using real time PCR. All data normalized to GAPDH = 10^6^. Experiments were performed in triplicate using cells from four different donors.

**Figure 4:**
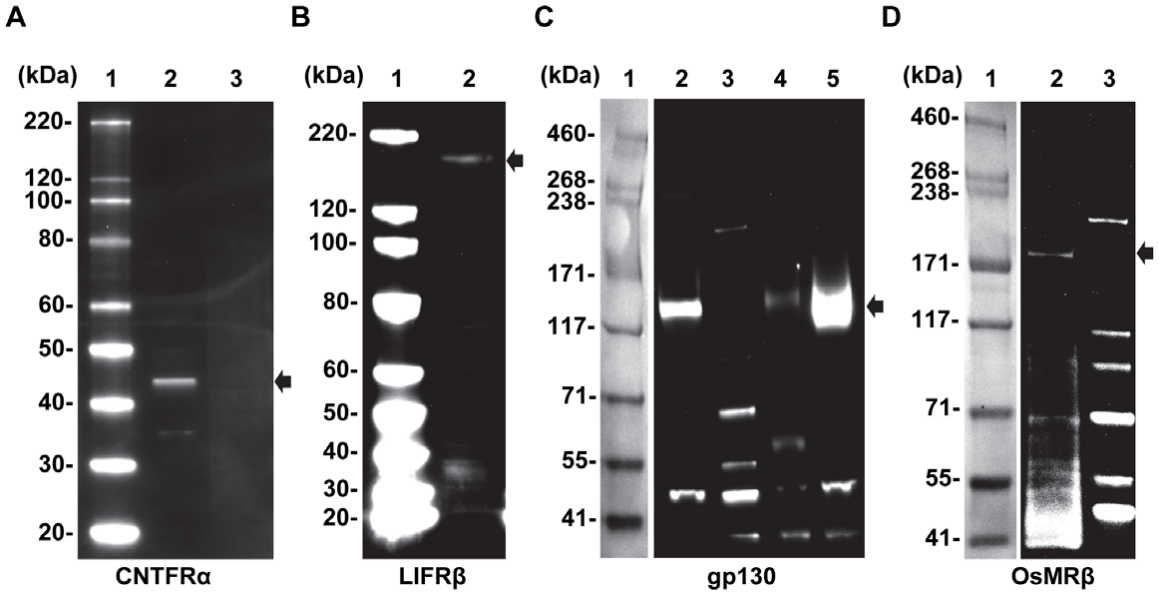
Constitutive expression of CNTF, CT1, OsM receptors in human RPE. 15 µg of enriched membrane proteins were electrophoresed and labeled with corresponding antibodies. Antibody specific bands (see arrows) for CNTFRα, LIFRβ, gp130 and OsMRβ are detected at approximately 41, 190, 130, and 200 kDa, respectively. **Panel A** (CNTFRα) - Lane 1: Magic Mark™ XP Western protein Standard; Lane 2: Brain (Human) membrane lysate-adult normal tissue; Lane 3: hfRPE membrane extract. **Panel B** (LIFRβ) - Lane 1: Magic Mark™ XP Western protein Standard; Lane 2: hfRPE membrane extract. **Panel C** (gp130) - Lane 1: HiMark™ Molecular Weight (HMW) Standard; Lane 2: hfRPE membrane extract; Lane 3: Magic Mark™ XP Western protein Standard; Lane 4: Hela whole cell lysate; Lane 5: NIH3T3 whole cell lysate. **Panel D** (OsMRβ) - Lane 1: HiMark™ Molecular Weight (HMW) Standard; Lane 2: hfRPE membrane extract; Lane 3: Magic Mark™ XP Western protein Standard.

We have previously shown by microarray analysis that multiple neurotrophic factors and receptors are expressed in native human fetal RPE cells, in primary cultures of fetal RPE, as well as in native human adult RPE cells [24]. In hfRPE, the relative expression levels of IL6 family members including CNTF, CT1, OsM, LIF and their receptors, LIFRβ, gp130, CNTFRα and OsMRβ were measured by qRT-PCR (Figure 3). Among the neurotrophic factors, LIF and CT1 are at least 1000-fold more highly expressed than CNTF and OsM. In addition, gp130 is the most highly expressed of the four receptor subunits (CNTFRα, LIFRβ, gp130, and OsMRβ). The presence of these four receptor subunits was further examined by immunoblot analysis (Figure 4). CNTFRα was under the detection limit (cells from 4 donors), but the presence of LIFRβ, gp130, and OsMRβ was confirmed showing antibody-specific bands at 190 kDa, 130 kDa, and 200 kDa.

**Figure 5:**
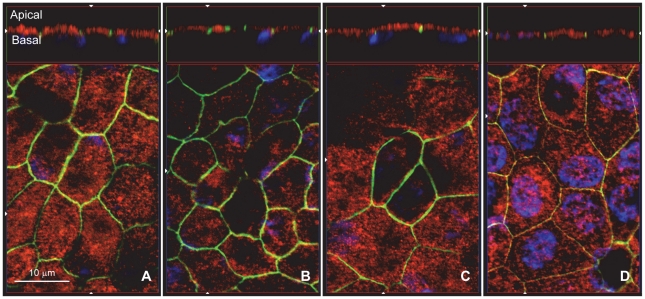
Immunofluorescence localization of CNTF, CT1, OsM receptors on hfRPE. Central part of each panel is an *enface* view of cell culture monolayer, shown as a single optical section obtained from a Z-stack. Nuclei were stained with DAPI (blue) and ZO-1 tight junction marker stained green. Images of the cross section through the Z-plane are shown at the top of each panel. CNTFRα (A, red), LIFRβ (B, red), gp130 (C, red) and OsMRβ (D, red) were detected mainly on the apical membrane of confluent monolayer of hfRPE.

We next localized CNTFRα, LIFRβ, gp130, and OsMRβ protein by immunofluorescence staining of confluent monolayers of hfRPE primary cultures (Figure 5). Primary antibodies were conjugated with different fluorophores to obtain image stacks along the Z-axis. Nuclei were stained with DAPI (blue), the tight junction marker ZO-1 in green, and the receptor subunits stained in red. The lower portion of each panel is an *enface* view of the monolayer, shown as an optical section obtained from the Z-stack. Each panel shows a hexagonal pattern of ZO-1 staining, typical of epithelia. CNTFRα appears as punctate staining visible throughout the cells. The top sides of each panel show a cross-section through the Z-plane. In these cross-sections, ZO-1 is a tight junction marker separating the apical and basolateral sides of the epithelial cells. Nuclei (blue) are located close to the basal side and serve as a marker to help define basal localization. CNTFRα, LIFRβ, gp130, and OsMRβ were all detected mainly on the apical membrane of hfRPE.

### Phosphorylation of STAT3 and ERK1/2

**Figure 6:**
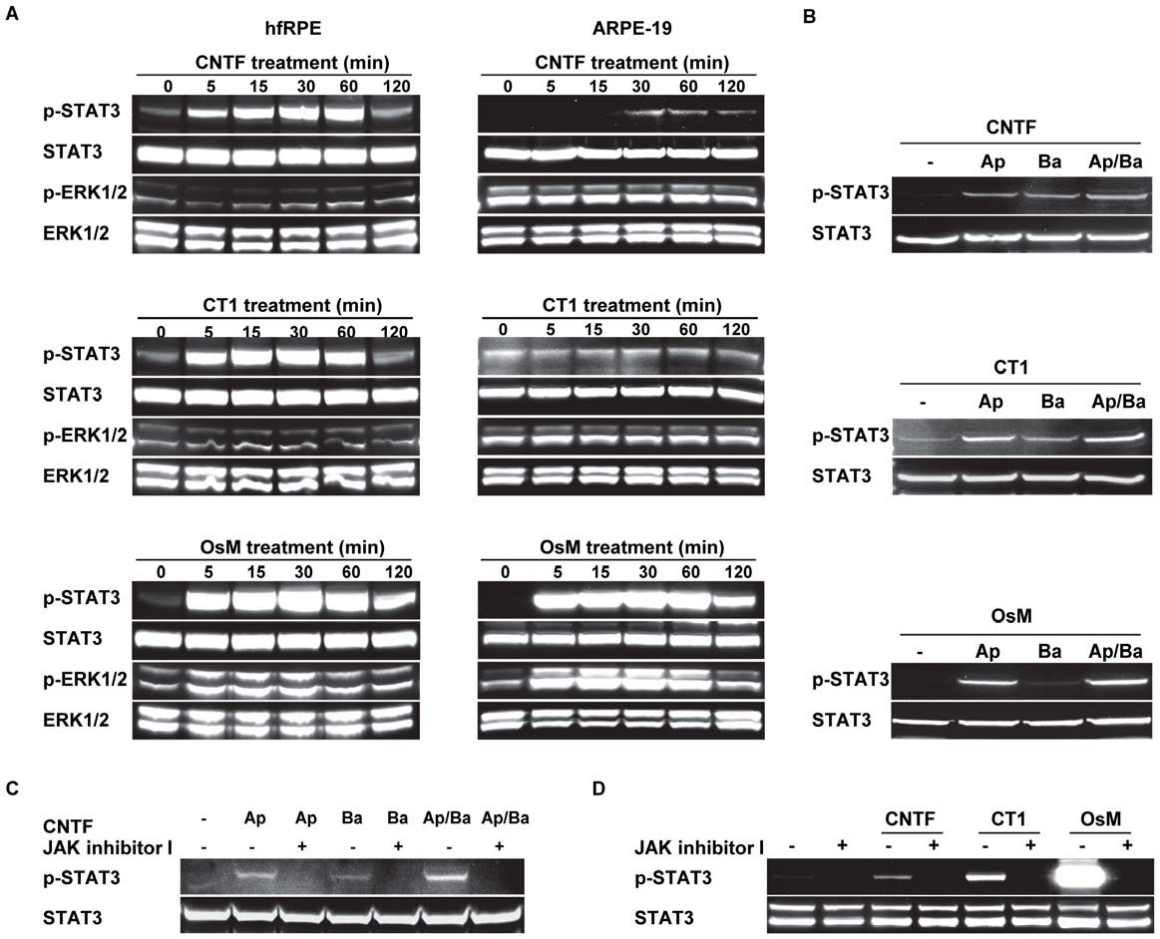
CNTF, CT1, and OsM induced activation of STAT3 and ERK1/2 in human RPE. **A.** Primary cultures of hfRPE and ARPE-19 were treated with CNTF, CT1, or OsM for a series of time points and the phosphorylation level of STAT3 and ERK1/2 were determined by Western blot analysis. **B.** CNTF, CT1, and OsM were added to the apical (Ap), basolateral (Ba), or both apical and basolateral (Ap/Ba) sides of confluent monolayers of hfRPE cells cultured on transwells for 30 minutes and phosphorylation levels of STAT3 were detected. **C.** Effects of JAK inhibitor I on CNTF induced phosphorylation of STAT3. Confluent monolayers of hfRPE cells were left untreated or treated with JAK inhibitor I (5 µM; both apical and basal sides) for 1 hour; 50 ng/ml of CNTF was added to the apical, basolateral, or both apical and basolateral side. Representative images from three separate experiments using cells from different donors. **D.** Effects of JAK inhibitor I on CNTF, CT1 and OsM induced phosphorylation of STAT3. Confluent monolayers of hfRPE cells were untreated or treated with JAK inhibitor I (5 µM; both sides) for 1 hour. 10 ng/ml of CNTF, CT1 or OsM was added to both apical and basolateral side and the phosphorylation levels of STAT3 were detected.

We treated hfRPE, as well as ARPE-19 cells, with CNTF, CT1, or OsM and measured the induced phosphorylation of STAT3 and ERK1/2. In hfRPE, an increase in STAT3 phosphorylation was detected as early as 5 minutes after treatment of CNTF, CT1, or OsM. The induced STAT3 phosphorylation peaked at 1 hour and lasted for 2 hours. OsM treatment also induced phosphorylation of ERK1/2 MAP kinase compared to the untreated control (at 0 minute) (Figure 6A, bottom left panel), which was not seen in cells treated with CNTF or CT1. In contrast, the CNTF-induced phosphorylation of STAT3 was delayed in ARPE-19 compared to hfRPE and was much weaker at all times points. CT1 treatment had no effect on STAT3 phosphorylation in ARPE-19 cells. In these cells, OsM treatment strongly increased the phosphorylation of STAT3 and ERK1/2 (Figure 6A, bottom right panel).

To examine the polarity of the CNTF, CT1, or OsM induced phosphorylation changes, we added each factor separately to the apical or basal baths and measured the induced STAT3 phosphorylation. Addition of CNTF to the apical or basal baths produced approximately equal STAT3 phosphorylation signals. Addition of CT1 to the apical bath always produced a stronger (≈1.5 fold) signal than addition to the basal bath. By comparison, the OsM induced STAT3 phosphorylation was ≈9-fold greater when added to the apical compared to the basal bath (Figure 6B). CNTF induced STAT3 phosphorylation was completely blocked when the cells were pretreated with JAK inhibitor I (5 µM added to both apical and basal baths) for 1 hour, regardless of whether CNTF was added to the apical or basal side of cells (Figure 6C), confirming the role of JAK kinase in mediating CNTF signaling in hfRPE cells. In another set of experiments, pretreatment with JAK inhibitor I (5 µM added to both apical and basal baths) for 1 hour completely blocked STAT3 phosphorylation induced by CNTF, CT1 or OsM added to both sides of the transwell (Figure 6D).

### RPE cell proliferation and survival

**Figure 7:**
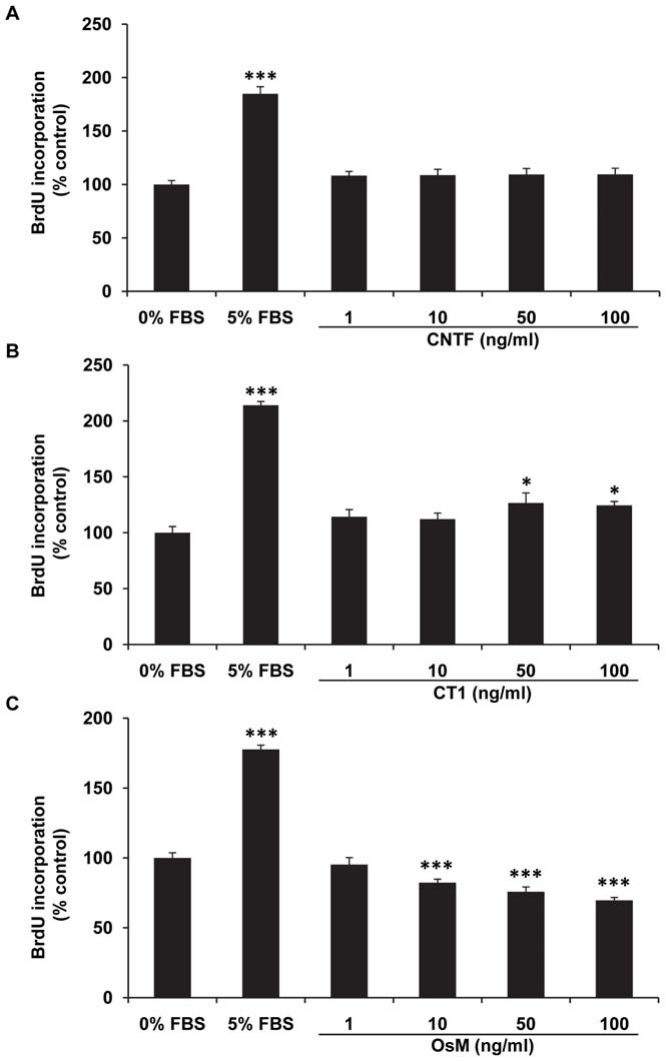
Dose responses of CNTF, CT1, and OsM - induced hfRPE proliferation. CNTF showed no significant effect on hfRPE proliferation, while CT1 significantly increased cell proliferation by 25% (50–100 ng/ml). In contrast, OsM produced a monotonic increase in its inhibitory effect on hfRPE proliferation between 1 and 100 ng/ml. There was a significant difference in inhibition between 1 and 10 ng/ml (*P* = 0.02) and between 10 and 100 ng/ml (*P* = 0.001). 5% FBS was used as positive control. (Summary data from two experiments using cells from different donors; * *P*<0.05, *** *P*<0.001 compared to 0% FBS negative control).

We next examined the effects of CNTF, CT1, and OsM on RPE cell proliferation and survival. The BrdU incorporation assay was used to assess hfRPE cell proliferation. The data summarized in Figure 7 show that CNTF had no significant effects within the tested range (1–100 ng/ml). By comparison, CT1 significantly increased hfRPE cell proliferation after 72 hours and the maximum stimulatory effect was 26.6±9.0% at 50 ng/ml. In contrast, OsM inhibited hfRPE proliferation monotonically from 1 to 100 ng/ml (Figure 7C).

**Figure 8:**
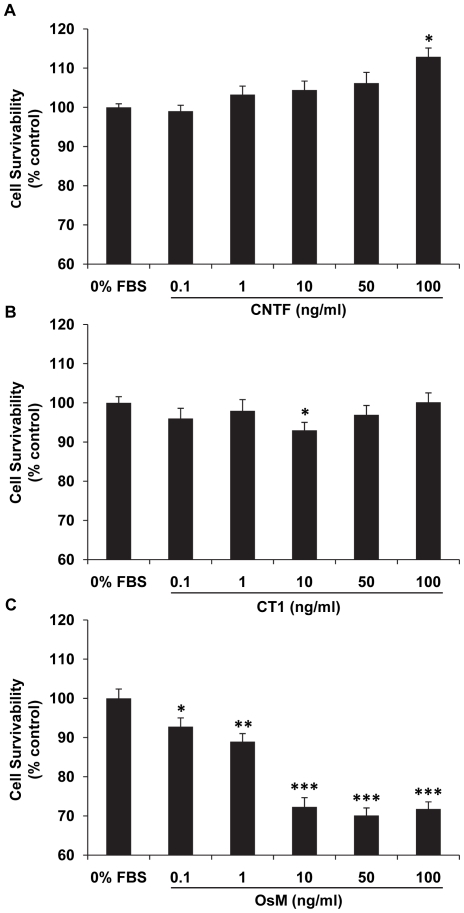
Dose responses of CNTF, CT1 and OsM effects on hfRPE survivability. CNTF showed significant protection on RPE cell survivability while OsM significantly decreased RPE cell survivability at concentrations starting from 0.1 ng/ml. (Summary data from experiments using cells from different donors; * *P*<0.05, ** *P*<0.01 *** *P*<0.001 compared to 0% FBS negative control).

Effects of CNTF, CT1, or OsM on cell survival were examined by the MTT assay. At a high dose (100 ng/ml) (12.9%; *P*<0.05, Figure 8A), CNTF induced an increase in hfRPE survival that is statistically significant. No significant changes in hfRPE survival were seen for CT1 except for a slight decrease at 10 ng/ml (−7%; *P*<0.05, Figure 8B). In contrast, OsM induced a significant dose-dependent decrease in hfRPE cell survival, from 0.1 to 10 ng/ml, with no further decrease between 10 and 100 ng/ml (Figure 8C).

### RPE cell phagocytosis

**Figure 9:**
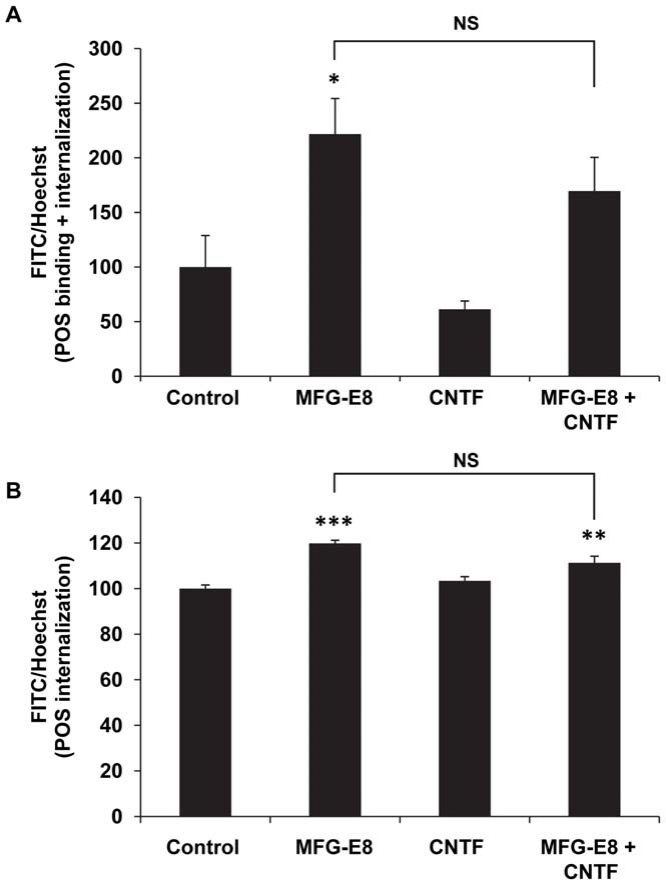
CNTF has no significant effect on hfRPE phagocytosis. Confluent monolayers of hfRPE received FITC-labeled POS for 4 hour with or without CNTF (50 ng/ml) and phagocytosis rate were quantified using relative FITC fluorescence intensity normalized to Hoechst 33342 nuclei stain. MFG-E8 (0.6 µM) was used as the positive control. Bar graph **A** show relative binding+internalization and bar graph **B** shows internalization only. Bars represent mean ± SEM with triplicate technical repeats. * *P*<0.05, ** *P*<0.01, *** *P*<0.001 compared to negative control. NS: no statistical significance compared to each other. Practically identical results were obtained in another experiment.

It has been shown that CNTF causes a shortening of photoreceptor outer segments [11], perhaps by activating CNTF receptors on the RPE apical membrane and increasing phagocytic activity. Figure 9 represent one of two independent experiments using bovine POS with triplicate technical repeats for each group. MFG-E8 was used as a positive control since it has been shown to increase phagocytosis by increasing the binding rate of photoreceptor outer segments [41]. CNTF (50 ng/ml) had no significant effect on phagocytosis rate compared to control and similar results were obtained at 20 and 100 ng/ml.

### CNTF alters polarized secretion of cytokines and neurotrophic factors from hfRPE

**Figure 10:**
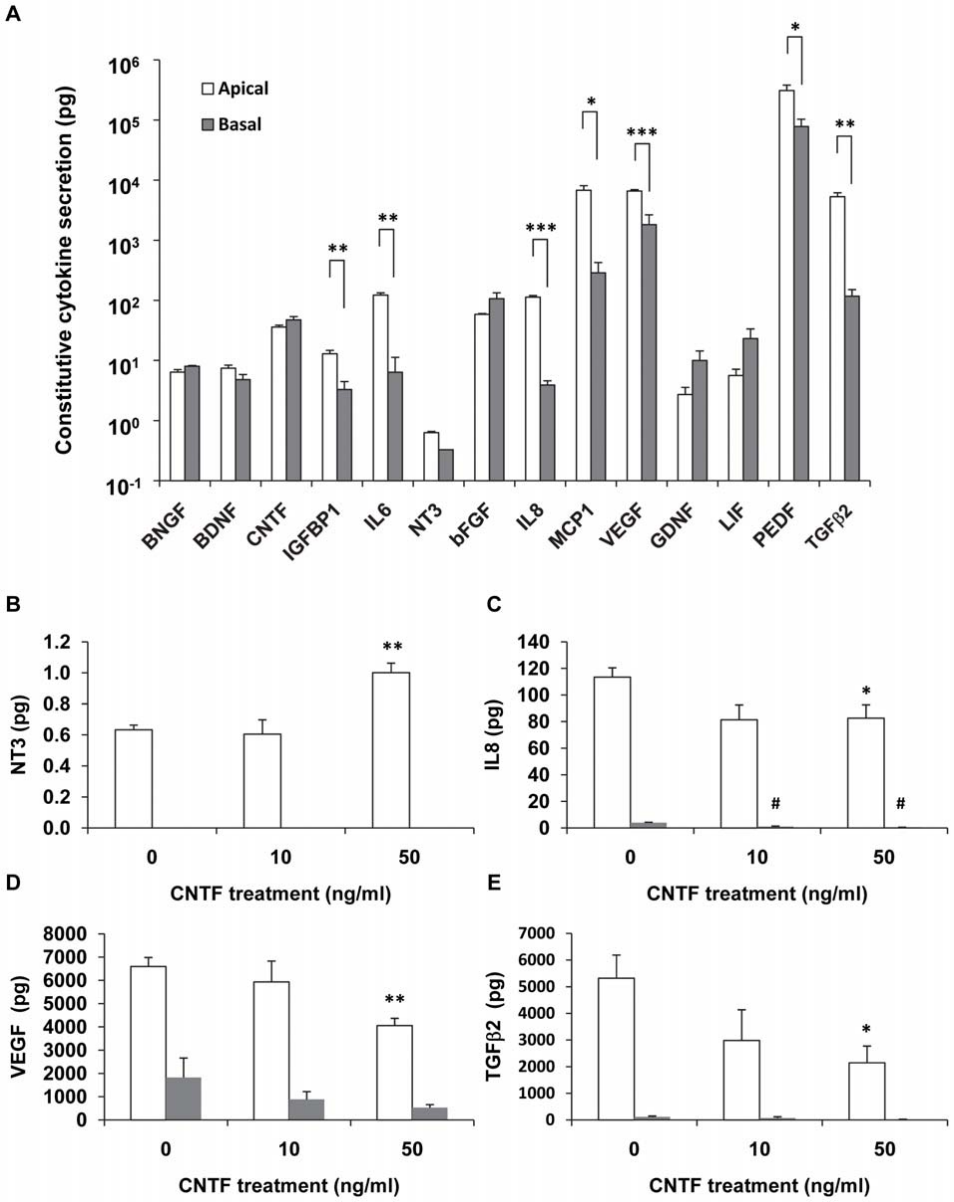
Effect of CNTF on polarized cytokine secretion from hfRPE. **A.** Constitutive secretion of cytokine and neurotrophic factors from confluent monolayer of hfRPE. * *P*<0.05, ** *P*<0.01, *** *P*<0.001 comparison between apical and basal compartment; **B–C.** CNTF-induced secretion changes of NT3, IL8, VEGF, and TGFβ2. * *P*<0.05, ** *P*<0.01, compared to the apical compartment of untreated control; #<0.05, compared to the basal compartment of untreated control.

hfRPE cells secrete a variety of growth factors, cytokines, and neurotrophic factors. Among the factors measured, PEDF, VEGF, MCP1, TGFβ2, IL6, and IL8 are preferentially secreted to the apical chamber at relatively high levels (0.1 to 1.0 ng) (Figure 10A). By comparison, bFGF, CNTF, LIF, BDNF, GDNF, and BNGF were secreted at much lower levels in a non-polarized fashion (Figure 10A). CNTF treatment had no significant effect on the secretion of bFGF, PEDF, IL6, MCP1, BDNF, GDNF, BNGF and IGFBP-1 (not shown). The constitutive secretion of NT3 is low (Figure 10A). However, its apical secretion was significantly increased by CNTF treatment (50 ng/ml) (Figure 10B). In striking contrast, the apical secretions of VEGF, IL8, and TGFβ2 were significantly inhibited by CNTF at 50 ng/ml (Figure 10C–E).

### CNTF increases fluid transport across RPE

**Figure 11:**
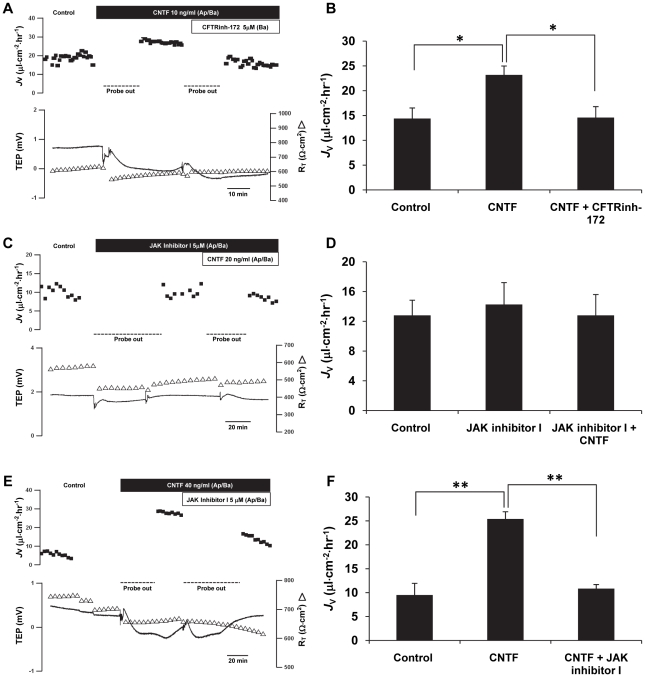
CFTRinh-172 inhibits CNTF-induced fluid transport across RPE. *J*_V_ is plotted as a function of time in the top trace and net fluid absorption (apical to basal bath) is indicated by positive values; TEP (-) and R_T_ (Δ) are plotted as function of time in the lower traces. For each set of experiments, left- hand panels (**A, C, E**), summary bar graphs are shown on the right-hand side (**B, D, F,** respectively) **A.** Addition of CNTF to the apical and basal bath increased *J*_V_ across monolayer of hfRPE; subsequent addition of CFTRinh-172 decreased *J*_V_ back to the baseline levels (n = 5). **C.** Pretreatment with JAK inhibitor I (5 µM) significantly inhibited the effect of CNTF on *J*_V_ increase (n = 3). **E.** Subsequent addition of JAK inhibitor I decreased *J*_V_ induced by CNTF (n = 3). * *P*<0.05, ** *P*<0.01 compared to each other.

Transepithelial fluid transport across confluent monolayers of hfRPE cultures was measured in a modified Ussing chamber (schematic in Figure 2). Figure 11A shows that addition of CNTF (10 ng/ml) to the apical and basal baths increased fluid transport (*J*_V_) by 8 µl⋅cm^−2^⋅hr^−1^ from the retinal to the choroidal side of the tissue. In six experiments, the mean *J*_V_ increased from 14.4±2.1 to 23.2±1.8 µl⋅cm^−2^⋅hr^−1^ (n = 6; *P* = 0.01) with no significant changes in steady-state transepithelial potential (TEP) and total tissue resistance (R_T_). Adding CFTRinh-172, a specific CFTR inhibitor (5 µM), to the basal side completely and reversibly abolished the CNTF-induced *J*_V_ increase (*P* = 0.02), indicating that the CNTF-induced *J*_V_ increase is predominantly mediated by CFTR chloride channels (Figure 11B). As shown in Figure 11C, pretreatment with JAK inhibitor I (5 µM) for 30 minutes had no effect on *J*_V_; in the presence of this inhibitor, CNTF had no effect on *J*_V_. In three such experiments, steady-state *J*_V_ was 12.8±2.0 µl⋅cm^−2^⋅hr^−1^ and this base line was not significantly altered by JAK inhibitor I (14.3±2.9 µl⋅cm^−2^⋅hr^−1^). In the continued presence of the JAK inhibitor I, CNTF failed to alter *J*_V_ (12.8±2.8 µl⋅cm^−2^⋅hr^−1^) (Figure 11D). In Figure 11E, we first activated the JAK/STAT3 pathway with CNTF and then added JAK inhibitor I. In three such experiments, CNTF increased *J*_V_ from 9.5±2.5 µl⋅cm^−2^⋅hr^−1^ to 25.4±1.5 µl⋅cm^−2^⋅hr^−1^ (*P* = 0.01), and further addition of JAK inhibitor I decreased *J*_V_ back to the pretreatment baseline (10.8±0.9 µl⋅cm^−2^⋅hr^−1^; *P* = 0.01; Figure 11F). These results demonstrate that the CNTF induced increase in *J*_V_ is mediated by CFTR following activation of the canonical JAK/STAT signaling pathway.

## Discussion

**Figure 12:**
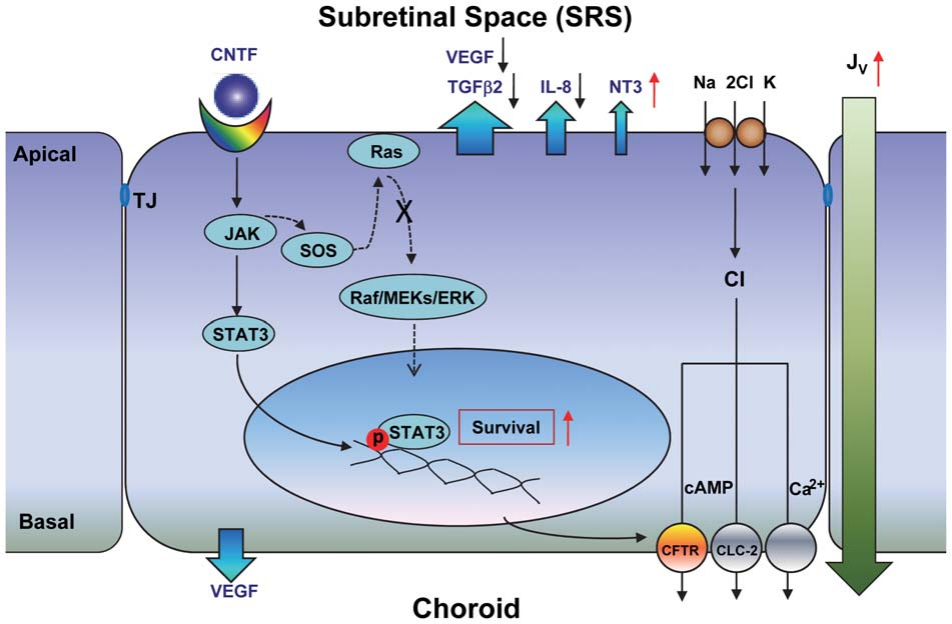
Schematic diagram of CNTF signaling in human RPE. Human RPE constitutively secretes a range of growth factors, cytokines, and neurotrophic factors, including VEGF, IL8, TGFβ2, CNTF, and NT3 (blue arrows) to the apical bath. In addition, retinal damage can increase CNTF release toward the subretinal space. CNTF binding to its receptors at the RPE apical membrane activates JAK/STAT3 signaling and promotes RPE survivability. It also decreases VEGF, IL8, and TGFβ2 secretion into the apical bath and increases apical NT3 secretion along with fluid absorption (*J*_V_), mediated by the activation of basolateral CFTR chloride channels.

We have shown that RPE cells express the neurotrophic factors, CNTF, CT1, LIF, and OsM, and their receptors, which when activated trigger the JAK/STAT3 signaling pathway in human RPE. CNTF receptor activation leads to profound changes in RPE cell physiology, via an increase in cell survival, an alteration in the polarized secretion of neurotrophic factors and cytokines, and an increase in fluid absorption across the RPE. Thus, the present experiments show how CNTF and related cytokines could influence the behavior of human RPE *in vivo* by leading to chemical/volume alterations in the subretinal space (SRS) and around Bruch's membrane (Figure 12). These changes may ultimately facilitate photoreceptor survival and function.

### CNTF signaling and RPE survivability

In many neuronal cells, CNTF induces phosphorylation of STAT3 as well as ERK1/2 MAP kinase [42], [43], [44], [45]. The presence of CNTFRα, LIFRβ, gp130, and OsMRβ, mainly on the apical membrane of RPE suggest that RPE cells can respond to changes of CNTF and related cytokines in the retina. Our results confirmed that CNTF, CT1, and OsM all induce STAT3 phosphorylation in human RPE. The fact that we failed to detect induction of STAT1 phosphorylation by CNTF treatment (not shown) suggests that the JAK/STAT3 pathway is the major signaling pathway mediating CNTF-induced changes in hfRPE. It has been shown previously that ARPE-19 cells are not responsive to CNTF because LIFRβ is not expressed in ARPE-19 cells (Song Y, et al. *IOVS* 2003;44:ARVO E-Abstract 390). However, we observed CNTF-induced STAT3 phosphorylation in ARPE-19 cells. The induction was very weak and only detectable after treatment for more than 30 minutes. These observations are another reflection of the differences between transformed cell lines (ARPE-19) and native or primary human RPE cultures [24].

Our results show that CNTF promotes human RPE cell survival but does not induce hfRPE proliferation. In contrast, CT1 induces hfRPE cell proliferation but does not promote survival. It is interesting that CNTF and CT1 share the same receptor complex of gp130 and LIFRβ, but have opposite effects on hfRPE proliferation and survival. It is quite surprising to see that OsM, another IL-6 family member, inhibits both hfRPE proliferation and cell survival. Human OsM, CNTF, and CT1 all bind to the gp130/LIFRβ receptors, while gp130/OsMRβ is specific for OsM [16], [46], [47]. Unlike CNTF and CT1, OsM not only induces STAT3 but also ERK1/2 phosphorylation in hfRPE cells. The possible involvement of the ERK1/2 signaling pathway may explain the inhibitory effects of OsM on hfRPE proliferation/survival.

### CNTF alters polarized secretion of cytokines by hfRPE

Early studies suggested that CNTFRα is not expressed on photoreceptor cells [48], [49], [50], [51], but recent reports indicate the expression of CNTFRα on photoreceptor precursors or even mature photoreceptors [6], [52], [53], [54], [55], [56], [57]. Although the localization of CNTFRα remains controversial [48], [49], [50], [51], there is evidence to indicate that photoreceptors are not directly responsive to CNTF [48], [49], [50]. It is thought that the neuroprotective effects of CNTF are mediated by other retinal cells, probably Müller cells, that directly respond to CNTF [48], [49] and release neurotrophic factors such as bFGF [9], [15]. CNTF-induced activation of the JAK/STAT3 pathway in RPE provides another possible mechanism for photoreceptor neuroprotection. CNTF is found within the microglial cells of the retina (Müller cells and astrocytes) as well as retinal pigment epithelium [58]. CNTF expression level increases in retinal damage [9], [10], [11] and part of this increase may come from the RPE which constitutively secretes CNTF (Figure 10A). *In vivo*, the subsequent activation of JAK/STAT3 signaling could change the polarized secretion of growth factors, cytokines, and neurotrophic factors to the SRS and thus provide protection for rod and cone photoreceptors.

Here we show that hfRPE constitutively secretes a host of neurotrophic factors such as CNTF, LIF, GDNF, BDNF, and BNGF (pg/ml levels). We found that CNTF stimulates the secretion of Neurotrophin-3 (NT3) from the apical side, but inhibits the secretion of VEGF, IL8, and TGFβ2. NT3 is a member of the neurotrophin family, which includes NGF (nerve growth factor), BDNF, NT3 and NT4/5 [59]. NT3 is a specific survival factor for large proprioceptive neurons found in the dorsal root ganglion [60] and has been shown to protect photoreceptors from light damage by stimulating Müller cells to produce FGF2 [61]. The increased release of NT3 through the RPE apical membrane may help protect photoreceptors against retinal degenerative disease, including AMD [62], [63], [64], [65], [66].

In contrast to the increase of NT3, secretion of VEGF, IL8, and TGFβ2 to the apical bath were substantially decreased after CNTF treatment. VEGF is a potent permeability factor and angiogenic growth factor whose levels increase significantly in various ocular diseases, such as proliferative diabetic retinopathy (PDR) and choroidal neovascularization (CNV) - the wet form of AMD [67], [68], [69], [70]. In CNV, the leading cause of blindness in the elderly, it is the leakage of serum from the invading blood vessels that causes disciform scar formation and blindness [71], [72], [73]. Therefore, the CNTF-induced decrease in VEGF secretion may be protective against the serum leakage from newly formed blood vessels that invade the SRS during CNV [72], [73], [74], [75]. IL8 is produced by macrophages and epithelia and its secretion is significantly increased in inflammation [76] and angiogenesis [74], [77]. TGFβ2 is critical in retinal development and involved in programmed cell death [78], [79]. TGFβ signaling may be involved in epithelial-mesenchymal-like transitions of RPE cells during PVR [80] and is associated with inflammation and viral infection in the retina [81]. Together, the inhibition of VEGF, IL-8, and TGFβ2 secretion by CNTF would inhibit serum leakage, angiogenesis, and inflammation in the retina during exudative AMD, PVR, and PDR.

### CNTF increases fluid absorption across human RPE

The CNTF-induced increase in fluid absorption may be protective in several disease states by increasing the activity of subretinal space cytokines or neurotrophic factors. For example, CNTF-induced decrease in SRS volume would increase the activity of apically secreted NT3 and therefore amplify its neuroprotective effects on nearby photoreceptors. In AMD, this increase could help offset the CNV-induced increase in fluid leakage from invading blood vessels [75]. The pro-inflammatory effects of IL8 and the proliferative/migration effects of TGFβ could also be mitigated if the decrease in these secretions were greater than the decrease in SRS volume.

### Summary

*In vivo*, the distal retina can be protected against damage or neurodegeneration by CNTF-induced activation of the JAK/STAT3 signaling pathway leading to: (1) an increase in RPE survivability; (2) increased secretion of protective factors to the subretinal space, which possibly act in conjunction with Müller cell secretion; and (3) an increase in fluid absorption out of the SRS via CFTR [19], which serves to maintain the normal close anatomical, mechanical, and physiological relationship of the photoreceptor outer segments and RPE apical membrane (Figure 12). Taken together, all of these functions serve to maintain the health and integrity of the neuroretina/RPE/choriocapillaris.
